# New Technology for the Use of Inhaled Nitric Oxide to Protect the Heart and Lungs during Operations with Cardiopulmonary Bypass

**DOI:** 10.17691/stm2020.12.5.03

**Published:** 2020-10-28

**Authors:** V.V. Pichugin, I.R. Seyfetdinov, M.V. Ryazanov, S.E. Domnin, A.B. Gamzaev, V.A. Chiginev, V.V. Bober, A.P. Medvedev

**Affiliations:** Professor, Department of Anesthesiology, Resuscitation and Emergency Medical Aid; Privolzhsky Research Medical University, 10/1 Minin and Pozharsky Square, Nizhny Novgorod, 603005, Russia; PhD Student, Department of Anesthesiology, Resuscitation and Emergency Medical Aid; Privolzhsky Research Medical University, 10/1 Minin and Pozharsky Square, Nizhny Novgorod, 603005, Russia; Associate Professor, Department of Hospital Surgery named after B.A. Korolyov; Privolzhsky Research Medical University, 10/1 Minin and Pozharsky Square, Nizhny Novgorod, 603005, Russia; PhD Student, Department of Anesthesiology, Resuscitation and Emergency Medical Aid; Privolzhsky Research Medical University, 10/1 Minin and Pozharsky Square, Nizhny Novgorod, 603005, Russia; Professor, Department of X-ray Endovascular Diagnostics and Treatment; Privolzhsky Research Medical University, 10/1 Minin and Pozharsky Square, Nizhny Novgorod, 603005, Russia; Professor, Department of Hospital Surgery named after B.A. Korolyov; Privolzhsky Research Medical University, 10/1 Minin and Pozharsky Square, Nizhny Novgorod, 603005, Russia; Assistant, Department of Anesthesiology, Resuscitation and Emergency Medical Aid; Privolzhsky Research Medical University, 10/1 Minin and Pozharsky Square, Nizhny Novgorod, 603005, Russia; Professor, Department of Hospital Surgery named after B.A. Korolyov Privolzhsky Research Medical University, 10/1 Minin and Pozharsky Square, Nizhny Novgorod, 603005, Russia

**Keywords:** inhaled nitric oxide, protection of the heart and lungs during surgery, surgery with cardiopulmonary bypass

## Abstract

**The aim of the study** was to evaluate the effectiveness of a new technology for the use of inhaled nitric oxide (NO) for the heart and lung protection during operations with cardiopulmonary bypass (СРВ).

**Materials and Methods.:**

The study included 90 patients who underwent heart valve surgery and combined procedures under CPB and pharmacological cardioplegia. Three groups were created: group 1 (control, n=30); group 2 (n=30) — NO inhalation (20 ppm) was conducted traditionally, that is, before and after CPB; group 3 (n=30) — NO inhalation was performed using a new technology — during the entire operation, with pulmonary artery perfusion and lung ventilation performed during CPB. Troponin I (cTn I) level, changes in the pulmonary function parameters, and clinical indicators were studied.

**Results.:**

Statistically significant lower levels of postoperative cTn I were registered in the patients of groups 2 and 3, at the same time, the levels were significantly lower in group 3 compared to group 2. The patients in group 1 (standardized anesthesia protocol) demonstrated an increase in the alveolar-arterial oxygen difference, an increase in intrapulmonary shunting, a decrease in blood oxygenation, and static lung compliance after СРВ. In both cases, NO inhalation retained the values of lung compliance and pulmonary oxygenating function after CPB, and in the patients of group 3, it also significantly reduced intrapulmonary shunting and alveolar-arterial difference after CPB. NO inhalation allowed a statistically significant decrease in the incidence of pulmonary dysfunction, acute respiratory failure, as well as the time of respiratory support in the ICU.

**Conclusion.:**

The developed technology for the use of inhaled NO in surgery with CPB provides a clinically marked protective effect on the heart and lungs. The effectiveness of the protective action of NO depends on the duration of its administration and is most pronounced when used during the entire operation, including CPB time.

## Introduction

The incidence of postoperative pulmonary complications after heart valve surgery is 8% [1], which is associated with the development of acute lung injury caused by cardiopulmonary bypass (CPB). The probable causes of the detected functional and morphological disorders in the lungs are inflammation, prolonged lung collapse, pulmonary ischemia and reperfusion, contact of blood with the circuit surface of the heart–lung machine, endotoxemia, surgical trauma, blood loss, and blood and blood substitute transfusion [2, 3]. The problem of intraoperative myocardial injury remains of current interest due to the critical impact of the early postoperative period on the clinical course, since it is this that causes acute heart failure in 15–20% of cardiac interventions and rhythm disturbances in 30% of these procedures [4, 5].

The available experimental and clinical data suggest the ability of nitric oxide (NO) to reduce ischemia/ reperfusion injury of the heart [6–9] and lungs [10–14]. The use of inhaled NO can be promising in protecting both the heart and lungs from injury during surgery with CPB. However, there has not been known until now any clinical study confirming the effectiveness of inhaled NO in preventing ischemia-reperfusion injury of the heart and lungs.

**The aim of this study** was to evaluate the effectiveness of a new technology of using inhaled nitric oxide in protecting the heart and lungs during surgery with cardiopulmonary bypass.

## Materials and Methods

The present study is a single-center, randomized, prospective study. The work was carried out at the Privolzhsky Research Medical University on the basis of the Specialized Cardiosurgical Clinical Hospital named after Academician B.A. Korolev from September 2016 to September 2019. The study was conducted in accordance with the Helsinki Declaration (2013) and approved by the Ethics Committee of the Specialized Cardiosurgical Clinical Hospital named after Academician B.A. Korolev.

### Patients.

 The study included 90 patients of both sexes who underwent heart valve surgery and combined procedures under cardiopulmonary bypass. After the randomization of the study (the envelope method), three groups were created: in group 1 (n=30) — control group — a standardized anesthesia protocol and CPB were used; in group 2 (n=30), NO inhalation (20 ppm) was conducted before and after CPB; in group 3 (n=30), NO inhalation (20 ppm) was conducted during the entire operation, including the time period of CPB, at the same time, perfusion of the pulmonary artery and lung ventilation were also performed during CPB.

The groups did not differ by the studied parameters. The demographic data of the patients, as well as their initial state, are presented in Table 1.

**Table 1:** Clinical features of the patients in the studied groups

Characteristics	Groups		
	1 (n=30), control	2 (n=30), NO before and after CPB	3 (n=30), NO during the entire operation
Sex (absolute number/%):			
male	12/40.0	13/43.4	13/43.4
female	18/60.0	17/56.6	17/56.6
Age (years) (М±σ)	54.1±1.4	57.9±1.0	58.6±1.4
Functional class, NYHA (absolute number/%):			
III	28/93.3	29/96.6	27/90.0
IV	2/6.6	1/3.3	3/10.0
Chronic heart failure stage (absolute number/%):			
IIA	27/90.0	27/90.0	23/76.6
IIB	3/10.0	3/10.0	6/20.0
III	0	0	1/3.3
Left (М±σ) ventricle ejection fraction (%)	56.0±1.5	58.3±1.2	53.4±1.4

All the patients underwent preoperative preparation, then they underwent surgical interventions depending on the type of heart valve lesions and combined interventions under normothermic bypass and crystalloid cardioplegia (Custodiol, Germany). The types of the performed operations are presented in Table 2.

**Table 2:** Types of performed operations (absolute number/%)

Types of operations	Groups		
	1 (n=30), control	2 (n=30), NO before and after CPB	3 (n=30), NO during the entire operation
One valve repair	13/43.3	9/30.0	5/16.6
Two valve repair	8/26.6	10/33.3	16/53.3
Three valve repair	2/6.6	5/16.6	2/6.6
Combined operations	7/23.3	5/16.6	7/23.3
Other operations	0	1/3.3	0

The main parameters of the surgery time period (CPB time and aortic cross-clamp time) in the patients of the three groups are presented in Table 3. The Newman–Keuls test (ANOVA) was used for comparison; no statistically significant differences were found between the groups.

**Table 3:** Main parameters of the operation time period in the patients of the studied groups (М±σ)

Parameter of the operation time period	Groups		
	1 control (n=30),	2 (n=30), NO before and after CPB	3 (n=30), NO during the entire operation
CPB time (min)	98.6±37.1	106.0±33.4	104.8±33.2
Aorta crossclapping time (min)	80.2±32.8	75.5±24.6	80.3±27.9

### Techniques of using inhaled nitric oxide.

 For inhalation therapy, the Tianox apparatus (RFNCVNIIEF, Russia) was used in all cases (see the Figure), which provides NO synthesis from the air directly during therapy, its delivery to the patient’s breathing system circuit, regulation and continuous monitoring of NO/NO_2_ concentration in the breathing mixture. Inhalation delivery of nitric oxide to the patients of groups 2 and 3 at the stages before and after CPB was performed to the inspiratory part of the breathing circuit. A special breathing system by Intersurgical (UK) was used for inhalation therapy. The gas flow of inhaled NO was 300–400 ml/min with a concentration of 23.70±0.62 ppm in the fresh oxygen flow, the total inhalation time being 2–4.5 h. The delivered inhalation mixture was monitored using an electrochemical NO/ NO_2_ analyzer. The average concentration of NO_2_ was 0.4–1.5 (0.90±0.07) ppm. The patients in group 3, simultaneously with inhalation of NO during CPB, underwent pulmonary artery perfusion with oxygenated blood, and low tidal volume ventilation [15, 16].

**Figure:**
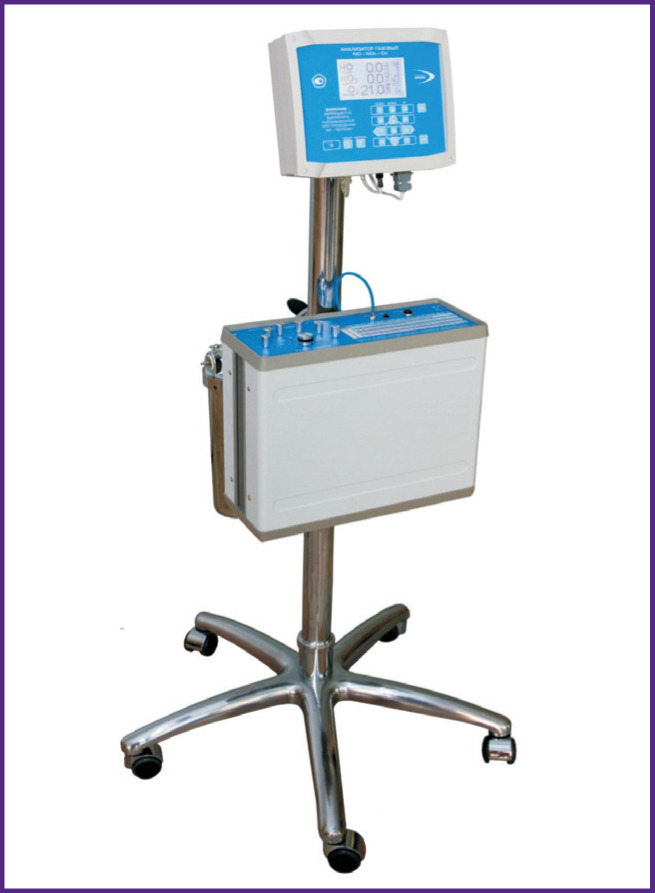
Tianox inhalation therapy apparatus (Russia)

### Research methods.

 To study the effectiveness of the protective effect of inhaled NO on the heart, the troponin I (cTn I) levels were determined at different stages: 1) when the patient entered the operating room (initially); 2) 12 h after surgery; 3) 24 h after surgery; 4) 48 h after surgery. The study of troponin I in plasma was performed using the PATHFAST cTn I test system, designed for diagnostics on the PATHFAST analyzer (Mitsubishi Chemical Medience Corp., Japan).

To assess the effectiveness of the protective effect of inhaled NO on the lungs, we studied the changes in the pulmonary function parameters: static lung compliance, the ratio of partial O_2_ tension and inspired O_2_ concentration (PaO_2_/FiO_2_), alveolar-arterial oxygen difference (AaPO_2_), growth rate of intrapulmonary blood shunting (F-shunt). The given indicators were studied at the following stages: 1) initially, after tracheal intubation and the beginning of artificial lung ventilation (ALV); 2) before CPB; 3) after completion of CPB; 4) at the end of the operation, before transferring the patient to the ICU. In addition to these indicators, the following ones were studied: the nature of the postischemic recovery of cardiac activity; the vasoactive-inotropic score (VIS) dynamics; the incidence of postoperative pulmonary dysfunction detected at impaired gas exchange (arterial hypoxemia, a decrease in the PaO_2_/FiO_2_ below 200 and a decrease in lung mechanics (decreased lung compliance)); the incidence of acute respiratory failure (ARF); the duration of respiratory support from the moment the patient enters the ICU; the length of patient stay in the ICU; the presence of other postoperative complications (acute heart failure, multiple organ dysfunction syndrome (MODS), acute cerebrovascular accident, bleeding); hospital mortality.

***Statistical analysis*** was performed using Microsoft Excel 2003, Biostatistics v. 4.03, and Statistica 6.0. The research results were processed in accordance with the rules of variation statistics. The distribution of the data was assessed using the Kolmogorov–Smirnov and Shapiro–Wilk tests. The arithmetic mean (M) and standard deviation (σ) were calculated for the data conforming to the law of normal distribution. The analysis of variance (ANOVA) was performed to check the reliability of differences between the mean values in the study groups by comparing the variances of these groups. When conducting pairwise multiple comparisons, the Newman–Keuls parametric test (ANOVA) was used for multiple comparisons [17]. Intergroup comparison was performed according to the χ^2^ criterion. The results of all tests below the critical value, i.e. p≤0.05, were considered statistically significant.

## Results

There was no statistically significant difference in the parameters of cardiac recovery after cardioplegia in the patients of the study groups. Thus, the frequency of spontaneous recovery after removing the clamp from the aorta was 76.6% in the patients of group 1, 80.0% in the patients of group 2, and 83.3% in the patients of group 3.

The effect of inhaled NO on the severity of myocardial contractile dysfunction was studied by the dynamics of the patients’ dependence on inotropic and vasopressor therapy and the level of cardiotonic support. No statistically significant difference in the VIS parameter after CPB and at the end of the operation in all the studied groups was detected.

The change in the level of cTn I at the stages of the postoperative period is presented in Table 4. The baseline preoperative troponin I levels did not have statistically significant differences in the patients of all study groups. After completion of the operation, a statistically significant increase of the cTn I level was observed in all groups of the patients: up to 2.62±0.20 ng/ml in group 1, up to 1.92±0.30 ng/ml in group 2, and up to 1.93±0.10 ng/ml in group 3. It should be noted that this level in the patients of groups 2 and 3 compared to group 1 was statistically significantly lower, while there was no difference between the patients in these groups.

**Table 4:** Dynamics of troponin I content in the postoperative period (ng/ml) (М±σ)

Groups	Initial value	After operation	12 h after operation	24 h after operation	48 h after operation
1 (control)	0.03±0.03	2.62±0.87*	3.41±1.73*	2.54±1.11^*^	1.65±0.63^*^
2 (NO before and after CPB)	0.01±0.00	1.72±0.95*^+^	2.47±0.88*^+^	2.29±0.81^*^	2.01±1.40^*^
3 (NO during the entire operation)	0.02±0.01	1.87±0.61*^+^	1.71±0.56*^+^	1.75±0.77*	0.80±0.40*^+#^

* Statistically significant differences compared with the initial study stage; ^+^ — with the control group at the same stage; ^#^ — with group 2 at the same stage.

Twelve hours after the operation, the patients of all the groups showed a statistically significant increase in the level of cTn I compared to the previous stage, while in the patients of groups 2 and 3 it was statistically significantly lower than in the patients of the group 1.

Twenty four hours after surgery, there was a further decrease in the level of cTn I in all groups of the patients. At this stage of the study, no statistically significant differences were found in the patients of the study groups.

Forty-eight hours after surgery, there was also a decrease in the level of cTn I in all groups of the patients: to 1.95±0.14 ng/ml in group 1, to 2.0±0.33 ng/ml in group 2, and up to 1.37±0.29 ng/ml in group 3. At this stage of the study, the level of cTn I was statistically significantly lower in the patients of group 3 compared to groups 1 and 2. There was no significant difference in this parameter between the patients of groups 1 and 2.

Thus, this clinical study confirmed the presence of a protective effect of inhaled nitric oxide on the myocardium, which manifested itself in lower levels of postoperative cTn I in the patients of groups 2 and 3. A comparative evaluation of the effectiveness of the technology of using inhaled NO showed its advantage in the event of continuous use of NO (in the patients of group 3) in combination with pulmonary artery perfusion and ventilation during CPB.

The changes in the pulmonary function parameters of the lungs in the groups such as alveolar-arterial oxygen difference, F-shunt index, PaO_2_/FiO_2_, static lung compliance are presented in Table 5. The initial data of all the studied functional parameters, as well as their dynamics before CPB did not have statistically significant differences between the groups.

**Table 5:** Changes in the pulmonary function parameters (М±σ)

Study stage	Groups		
	1 (n=30), control	2 (n=30), NO before and after CPB	3 (n=30), NO during the entire operation
***Alveolar-arterial oxygen difference***			
Beginning of operation	177.4±65.0	204.7±62.4	210.2±65.9
Before CPB	186.9±57.8	191.8±66.6	212.6±90.6
After CPB	210.9±74.9	233.0±78.6	214.4±79.5
After operation	229.8±67.0*	278.1±82.4*	238.5±79.0
***F-shunt***			
Beginning of operation	2.73±0.81	2.67±0.84	2.74±0.87
Before CPB	2.79±0.50	2.50±0.87	2.75±1.21
After CPB	3.85±1.03*	3.04±1.04^+^	2.82±1.05^+^
After operation	4.23±0.82*	3.75±1.09*^+^	3.13±1.05^+#^
***PaO*_2_/*FiO*_2_**			
Beginning of operation	394.3±100.2	446.8±83.2	434.6±86.2
Before CPB	375.9±72.3	457.1±97.2	432.8±123.7
After CPB	325.6±102.6*	404.9±112.1	431.2±104.9
After operation	283.0±98.7*	344.8±114.8*	395.7±104.0^+^
***Lung compliance***			
Beginning of operation	55.5±7.9	58.6±14.1	51.8±16.4
Before CPB	54.0±8.8	59.3±14.1	50.8±17.6
After CPB	49.7±9.6*	59.5±14.0^+^	54.8±17.2
After operation	47.1±8.7*	56.5±14.7^+^	57.3±19.4^+^

* Statistically significant differences compared with the initial study stage; ^+^ — with the control group at the same stage; ^#^ — with group 2 at the same stage.

After cardiopulmonary bypass, no statistically significant changes in the AaPO_2_ index in the patients of all studied groups were noted. At the end of the operation, this indicator increased with statistical significance by 29.6 and 35.8% in groups 1 and 2, respectively (compared to the initial data). At this stage of the study, in intergroup comparison, the AaPO_2_ index was higher with statistical significance in the patients of group 2 compared with groups 1 and 3. There were no statistically significant changes in the AaPO_2_ index in the patients in group 3 at all stages of the study.

The changes in the F-shunt index after CPB revealed a statistically significant increase from the initial one (by 40.7%) in the patients of group 1 with no significant changes in groups 2 and 3. At this stage, the values of the indicator in groups 2 and 3 were statistically significantly lower than in group 1. No significant difference was found in groups 2 and 3.

At the end of the operation, an increase in the F-shunt index was noted in group 1 by 55.5% from the

baseline, in group 2 — by 42.3% and in group 3 — by 14%, the changes in the patients of groups 1 and 2 being statistically significant. At this stage of the study, in intergroup comparison, the F-shunt values in the patients of group 3 were statistically significantly lower than in the patients of groups 1 and 2. There was no statistically significant difference in the F-shunt values between groups 1 and 2. It is important to underline that there were no statistically significant changes in the F-shunt index in the patients of group 3 at all stages of the study.

The analysis of the PaO_2_/FiO_2_ indices after CPC revealed a statistically significant decrease in their values compared to the baseline in group 1 by 17.4%, while there were no significant changes in the patients of groups 2 and 3. In the intergroup comparison, no statistically significant differences were found between groups 2 and 3.

At the end of the operation, there was a decrease in PaO_2_/FiO_2_ in group 1 by 25.4% from the baseline, in group 2 — by 22.8%, and in group 3 — by 8.9%, and these changes were statistically significant in the patients of groups 1 and 2. In the intergroup comparison, there was no statistically significant difference between the patients of groups 1 and 2 as well as in groups 2 and 3, while at this stage the PaO_2_/FiO_2_ values in group 3 were statistically significantly higher than in group 1. It should be underlined that there were no statistically significant changes in the PaO_2_/FiO_2_ index in group 3 at all stages of the study.

The analysis of lung compliance after cardiopulmonary bypass revealed a statistically significant decrease by 10.4% from the baseline in the patients of group 1, while in groups 2 and 3 its changes were statistically insignificant. The lung compliance values in the patients of group 2 at this stage of the study were statistically significantly higher than in the patients of group 1. When comparing groups 2 and 3, no statistically significant differences were found.

After surgery, lung compliance significantly decreased by 15.1% from the initial value in group 1. In the patients of groups 2 and 3, the decrease was insignificant and less than in group 1. It is important to underline that no statistically significant changes in this parameter were observed in the patients of groups 2 and 3 at all stages of the study.

Thus, our work demonstrated that with a standardized perioperative care protocol, after cardiopulmonary bypass, the pulmonary function parameters worsen, namely, an increase in the alveolar-arterial oxygen difference, an increase in intrapulmonary blood shunting, a decrease in blood oxygenation (PaO_2_/FiO_2_), a decrease in static lung compliance. The use of NO inhalation before and after CPB or NO inhalation during the entire operation (in combination with perfusion of the pulmonary artery and ventilation during CPB) made it possible to effectively maintain the values of lung compliance and pulmonary oxygenating function. There were no statistically significant changes in the parameters of lung compliance and PaO_2_/FiO_2_ depending on the type of NO inhalation (before and after CPB or during the entire operation). The use of the method of continuous NO inhalation during the operation (in combination with pulmonary artery perfusion and ventilation during CPB) statistically significantly reduced the growth of intrapulmonary shunting and alveolar-arterial difference after completion of CPB.

All the patients included in the study were discharged from the clinical departments of the hospital in a satisfactory state with clinical improvement in the underlying pathology after cardiac surgery under CPB. The diagnosis of pulmonary dysfunction in the early postoperative period was carried out on the basis of disturbances in lung mechanics (decrease in static lung compliance) and gas exchange disorders (development of arterial hypoxemia) after CPB. Gas exchange disorders were diagnosed on the basis of a decrease in the PaO_2_/FiO_2_ value below 200. The development of pulmonary dysfunction and acute respiratory failure was statistically significantly lower in the patients of group 3 compared with groups 1 and 2. Intergroup comparison was performed according to the χ^2^ criterion. Among the patients of group 1, pulmonary dysfunction was observed in three people (10.0%), two of them (6.6%) developed ARF. In the patients of group 2, the development of pulmonary dysfunction was observed in two patients (6.6%), one of them (3.3%) developed ARF. There was no development of postoperative pulmonary dysfunction among the patients in group 3.

Comparative assessment of the time of respiratory support in the ICU in the three groups was carried out using the χ^2^ criterion. A statistically significant earlier activation was demonstrated in the patients of group 3 compared to groups 1 and 2, the activation times being statistically significantly faster in the patients of group 2 compared with group 1. In the first 8 h after surgery, only 17 patients (56.6%) were activated in group 1, while in groups 2 and 3 — 22 (73.3%) and 26 (86.6%), respectively. There were no lethal outcomes in all three study groups.

Comparative assessment of the incidence of postoperative complications in the patients of all groups (comparison was carried out according to the χ^2^ criterion) did not reveal a significant lower incidence of complications in the patients of group 3 compared with the others. At the same time, no statistically significant differences were found in the incidence of postoperative complications in the patients of group 2 compared to group 1. This indicator in group 1 was 16.6%, of which ACF developed in 6.6% of cases, ARF in 6.6%, and MODS in 3.3%. In group 2, complications developed in 10.0% of cases, equally in the number — 3.33% each. In the patients of group 3, the development of a postoperative complication (coagulopathic bleeding) was noted in only one case (3.3%), no connection was found between its development and the technologies used.

Thereby, inhalation therapy with nitric oxide during operations on the heart valves according to our proposed method had a marked effect on the early postoperative period, which manifested itself both in a decrease in the incidence of postoperative pulmonary dysfunction and acute respiratory failure, and in a decrease in the time of patient activation after surgery.

## Discussion

There are very few clinical studies on the use of nitric oxide to improve myocardial protection during cardiac surgery under cardiopulmonary bypass and cardioplegic cardiac arrest and all of them are devoted to the introduction of the preparation into the extracorporeal circulation circuit. The first publication by Gianetti et al. appeared in 2004 [18]. The authors investigated the effect of inhaled NO (20 ppm) administered for 8 h during and after cardiopulmonary bypass and came to the conclusion that NO inhaled at a low concentration can reduce the release of markers of myocardial damage and counteract the development of subclinical left ventricular dysfunction during and immediately after cardiopulmonary bypass. All subsequent studies [19–21] also described the introduction of gaseous NO into the CPB circuit and confirmed its cardioprotective effect which was associated with lower VIS levels and cTn I and CK-MB cardiac-specific blood markers. We did not find any clinical studies on other technologies for the use of inhaled nitric oxide during operations with bypass surgery in the available literature.

We used two alternative technologies for the application of NO: with the traditional one, we inhaled NO (20 ppm) through a ventilator before and after CPB; using the other technology (proposed by us), inhalation was performed throughout the entire operation, including the CPB time period, while during CPB, perfusion of the pulmonary artery and ventilation of the lungs were performed. With both technologies, the drug entered the blood by inhalation through the alveolar membrane of the lungs and it did not contact with the synthetic membrane of the oxygenator, the features of interaction with which have not yet been studied. Thus, the physiological inhalation way of the delivery of a gaseous agent is an indisputable benefit of the technology we use.

The protective effect of NO inhalation on the heart is known to be associated with an increase in the amount of NO metabolites in the blood to the level sufficient to reduce ischemia-reperfusion injury to the myocardium [10]. It is this that can explain a higher protective effect on the myocardium in the patients of group 3, when NO was used for a longer time (throughout the entire operation, for 3.5–4.5 h), compared with group 2, where it was used only before and after CPB (for 1.0–1.5 h).

The results of our study of the pulmonary function parameters in the patients of the control group demonstrate a statistically significant increase in the alveolar-arterial oxygen difference, an increase in intrapulmonary shunting, and a decrease in oxygenation and lung compliance after cardiopulmonary bypass. The functional changes in the lungs in this group of patients presented in the study explain the appearance of pulmonary complications in the early postoperative period, namely the development of postoperative pulmonary dysfunction in 10.0% of the patients with the formation of acute respiratory failure in 6.6% of cases. Apparently, the development of postoperative pulmonary complications results from ischemiareperfusion lung injury. The received data on lung injury during CPB are consistent with the results of the previous studies [22–24].

The patients of group 2 showed the preservation of the AaPO_2_ and lung compliance values during the entire operation, as well as a decrease in the incidence of early postoperative complications, which indicates the presence of a protective effect of this technology on the lungs. Apparently, here we should consider the effect of pharmacological preconditioning of lung tissue induced by NO inhalation [11–13].

In the patients of group 3, when ventilation and blood delivery to the lungs are performed during the entire operation including the CPB time period, well-ventilated areas are constantly preserved in the lungs, which enables effective action of exogenous NO. Thereby, the intraoperative injury to the lung tissue is reduced. This is confirmed by high preservation of pulmonary function parameters (AaPO_2_, F-shunt, PaO_2_/FiO_2_, lung compliance) and the absence of early postoperative complications associated with pulmonary function.

This technology has been successfully implemented in clinical practice. A Russian patent was obtained for its development.

## Conclusion

The developed technology for the use of inhaled nitric oxide including its delivery during the entire operation with cardiopulmonary bypass together with pulmonary artery perfusion and ventilation of the lungs has a clinically marked protective effect on the heart and lungs.
